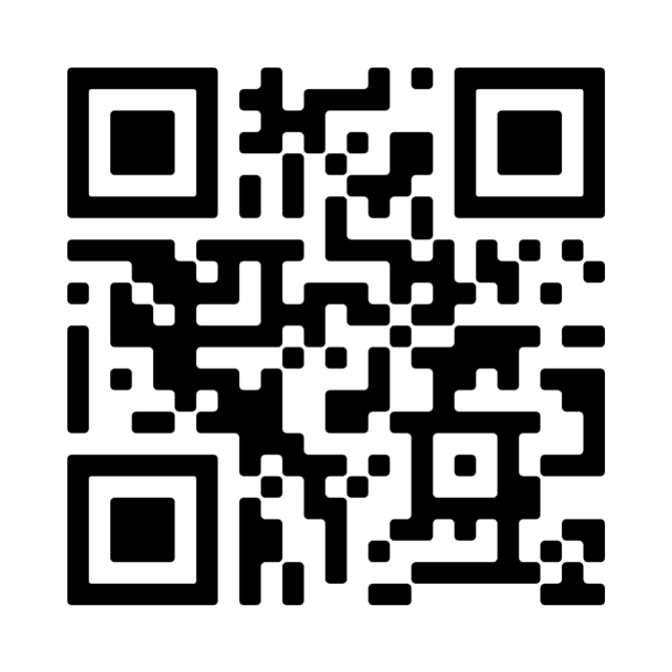# Laparoscopic resection rectopexy (RRP) combined with mesh sacrocolpopexy (SCP) for obstructed defecation syndrome with pelvic organ prolapse in an interdisciplinary approach

**DOI:** 10.52054/FVVO.16.2.017

**Published:** 2024-06-28

**Authors:** C Rudroff, S Ludwig

**Affiliations:** Department of Visceral Surgery and Functional Surgery of the Lower Gastrointestinal Tract (UGI), Clinic for General and Visceral Surgery, Evangelisches Klinikum Köln Weyertal gGmbH, Weyertal 76, 50931 Cologne, Germany; Department of Obstetrics and Gynecology, Division of Urogynecology and Pelvic Reconstructive Surgery, University Hospital of Cologne and Medical Faculty Cologne, Kerpener Str. 34, 50931 Cologne, Germany

**Keywords:** obstructed defecation syndrome (ODS), pelvic organ prolapse (POP), resection rectopexy (RRP), sacrocolpopexy (SCP), polyvinylidene-fluoride (PVDF), Interdisciplinary

## Abstract

**Background:**

Obstructive defecation syndrome (ODS) is frequently associated with pelvic organ prolapse (POP) and compromises the quality of life in affected patients. In cases conservative treatment fails surgical therapy is required.

**Objectives:**

The video case study combines a laparoscopic resection rectopexy (RRP) with a mesh sacrocolpopexy (SCP) in an interdisciplinary surgical approach.

**Materials and Methods:**

In this video an 86-year-old woman with ODS and POP, suffering from a dolichocolon with rectal intussusception, an apical prolapse after total hysterectomy 1990, and occasional stress urinary incontinence underwent interdisciplinary laparoscopic surgery. A tubular anterior rectal and sigmoid resection with suture rectopexy as in a resection rectopexy (RRP) was combined with a sacrocolpopexy (SCP) using a synthetic mesh.

**Main outcome measures:**

Surgical outcome including postoperative morbidity, functional bowel evacuation, and POP reconstitution as in POP-Q score after surgery were documented.

**Results:**

No intra- or postoperative complications occurred. At 6 months follow-up clinical outcomes for ODS, bowel dysfunction, and faecal control were improved. Anatomical outcome for POP and stress urinary incontinence symptoms were corrected.

**Conclusions:**

We report a promising interdisciplinary surgical approach as a single treatment option for the complex medical condition of women suffering from ODS and POP combining laparoscopic RRP with SCP. This surgical approach proved to be feasible, safe, and effective.

## Learning objective

This video shows a new and interdisciplinary surgical approach for the treatment of complex defecation problems with generalised pelvic floor defects.

It presents laparoscopic resection rectopexy in combination with a sacrocolpopexy in an interdisciplinary setting with visceral surgery and gynaecology. The surgical approach was feasible and effective in the presented case - offering affected women an interdisciplinary surgical procedure in a single treatment option.

## Introduction

Obstructive defecation syndrome (ODS) is a disturbed defecation process due to a protrusion of the lower rectum (rectocele) and a telescoping within the rectum (intussusception) (Andromanakos et al., 2006; Hedrick and Friel, 2013; Yagi et al., 2018). Patients exert pressure and manual manipulation to evacuate the rectum (Costilla and Foxx-Orenstein, 2014). ODS affects approximately 10%–25% of the population, most commonly in women, with the condition often associated with pelvic organ prolapse (POP) (Murad-Regadas et al., 2012; Wu et al., 2014; Guzman Rojas et al., 2016). Affected patients suffer from frustration due to their disturbed defecation, compromising their quality of life. Surgery is required in cases where conservative treatment options fail (Glazener et al., 2014; Hagen and Stark, 2011). Almost 20% of the women require surgery during their lifetime (Smith et al., 2010; Sanses et al., 2016). To date, the treatment of ODS with POP has been characterised by individual surgical approaches (Grimes et al., 2016; Lee et al., 2020) and surgical treatment options - particularly an interdisciplinary approach for evidence-based counselling of women with ODS and POP - is missing.

This video presents a laparoscopic surgical approach as an interdisciplinary single treatment option for the complex medical condition of women suffering from ODS and POP.

## Patients and methods 

This video presents a laparoscopic approach combining a rectal reconstruction with a resection rectopexy (RRP) and a sacrocolpopexy (SCP) with a polyvinylidene-fluoride (PVDF) mesh performed as an interdisciplinary procedure. Consent from the patient was taken for the use of the images and videos for publication.

An 86-year-old woman suffering from an obstructed defecation syndrome and a pelvic organ prolapse with occasional stress urinary incontinence was included in this case study. The dolichocolon was diagnosed by colon contrast x-ray prior to surgery (Figures 1 and 2). Bowel function was gathered using the Altomare score for ODS symptoms, the rectal toxicity score for bowel dysfunction, and the Wexner incontinence score for faecal control (González-Argenté et al., 2001; Altomare et al., 2008). For the POP the pelvic organ prolapse quantification score (POP-Q) was assessed (Table I).

**Figure 1a:**
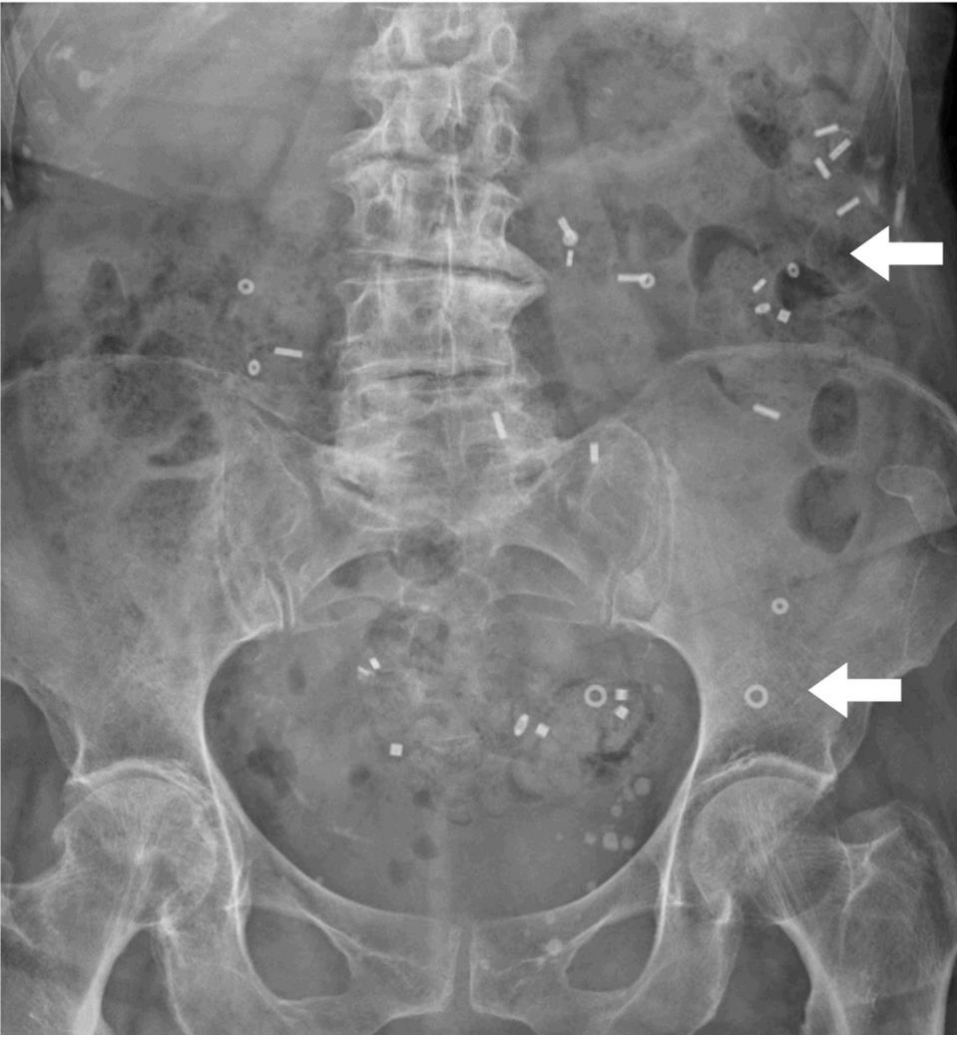
Radiologic imaging prior to surgery. The colonic transit time was with half of the markers in situ slightly elevated (48 hours). Interestingly, all markers were stuck in the left hemicolon (white arrows).

**Figure 1b:**
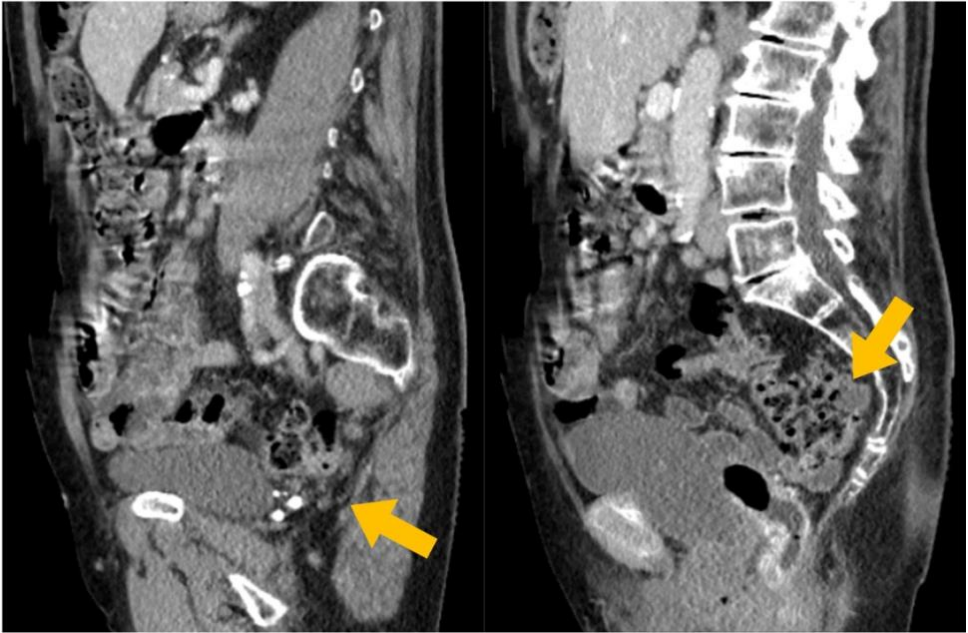
Radiologic imaging prior to surgery. The abdominal CT scan showed the relevant and, in most part, retrorectal elongation of the sigmoid colon in the two sagittal pictures (yellow arrows).

**Figure 1c:**
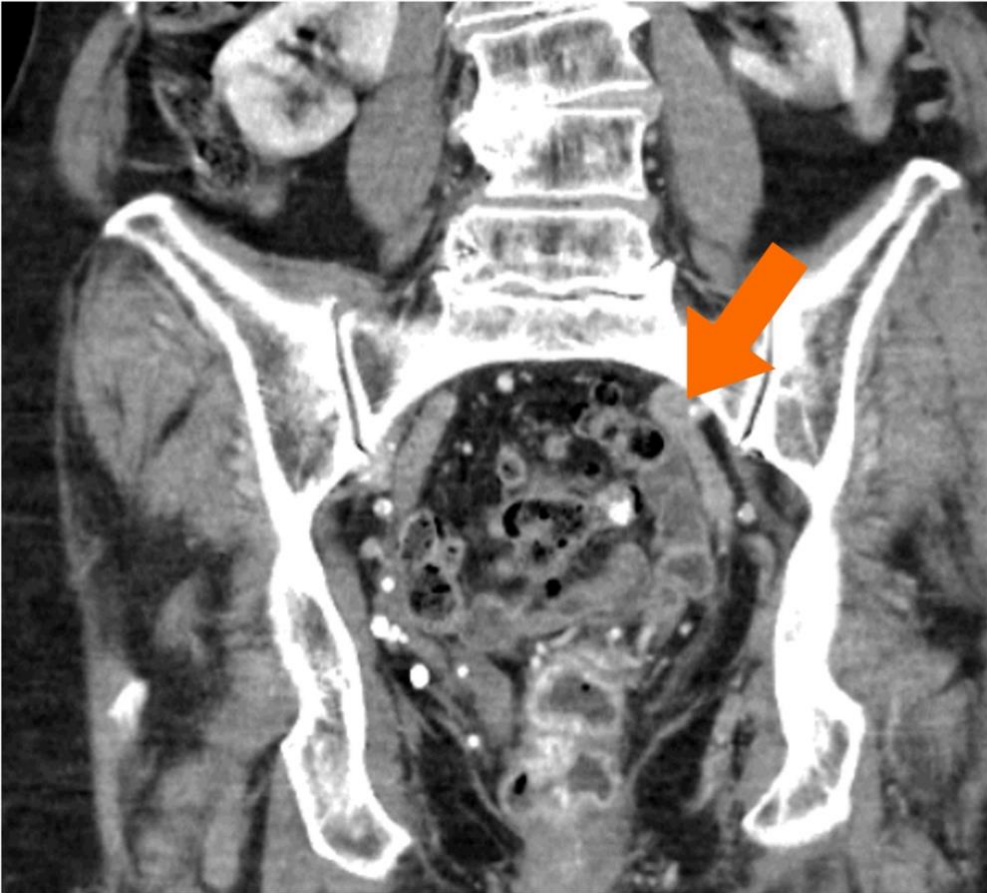
Radiologic imaging prior to surgery. The coronary view of the CT scan shows the crowed small pelvis filled with filled colon windings (orange arrow).

**Figure 2:**
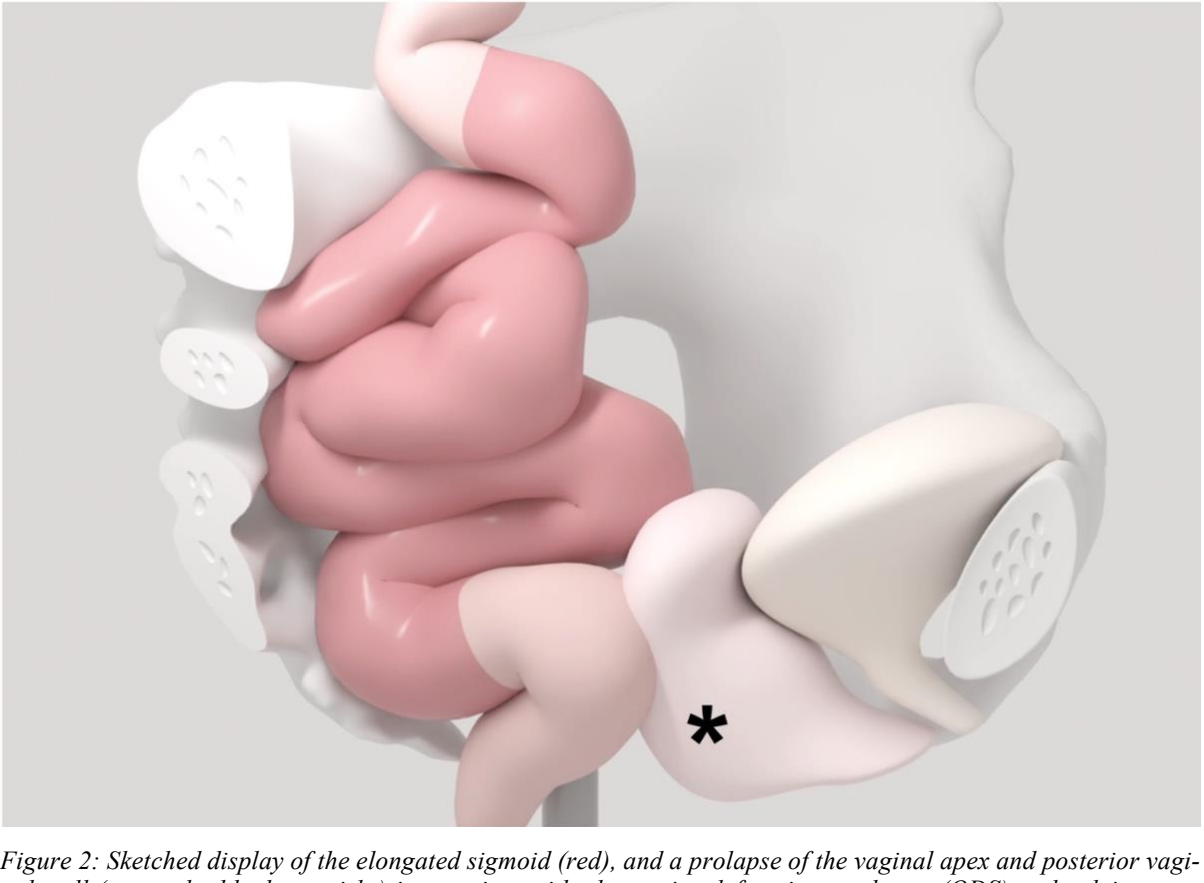
Sketched display of the elongated sigmoid (red), and a prolapse of the vaginal apex and posterior vaginal wall (rectocele, black asterisks) in a patient with obstructive defecation syndrome (ODS) and pelvic organ prolapse (POP) before surgery.

**Table I:** Patient´s characteristics, surgical details, and clinical scores before and after surgery.

Age in years at surgery	86	
ASA	3	
BMI kg/m^2^	22	
Operating time (minutes)	223	
Duration of hospital stay (days)	8	
CDC (0-5)	0	
Clinical Scores	preoperative	postoperative
Altomare score (n/30)	7	0
Rectal Toxicity score (n/32)	11	0
Wexner incontinence score (n/20)	14	2
POP-Q stage	2	0

For the laparoscopic procedure under general anaesthesia, the patient was maintained in dorsal lithotomy with the head tilted 18°. The CO2 pneumoperitoneum was established according to institutional standards through the first umbilical trocar placed in the open Hasson technique (Hasson, 1971). Three trocars were placed in the lower and middle right and the left lower abdominal quadrant. The procedure started with the preparation of the rectum down to the pelvic floor by opening the retro-rectal and the rectovaginal space to ensure the complete mobilisation of the rectum down to the pelvic floor. From the aboral resection margin, a tubular resection of the anterior rectum and the elongated sigmoid with its functional kinking was performed. The bowel continuity was reconstructed with an end-to-end descendo rectostomy with a circular stapling device (29-mm Endoscopic Curved Intraluminal Stapler, Johnson & Johnson).

**Figure 3:**
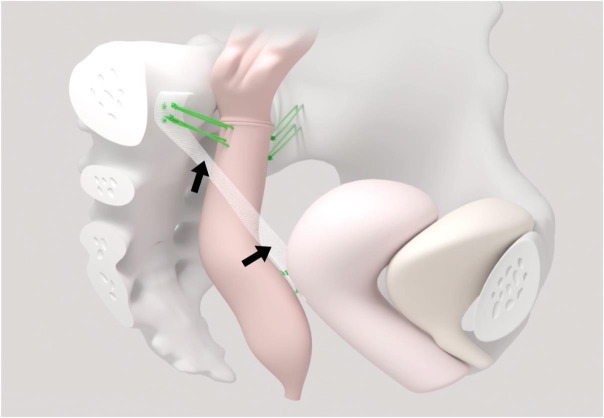
Postoperative situs after laparoscopic resection rectopexy with mesh sacrocolpopexy (black arrows) for obstructed defecation syndrome (ODS) and pelvic organ prolapse (POP). The rectopexy (two green sutures) is done on the right and left sides of the pelvis.

Next, the apical fixation of the middle compartment was performed unilaterally on the right patient side. The mesh for the sacrocolpopexy was fixed to the vaginal vault with two absorbable sutures (Vicryl 2-0, Johnson & Johnson) and one non-absorbable suture (Ethibond 2-0, Johnson & Johnson). The mesh (FEG Textiltechnik mbH, Aachen, Germany; DynaMesh, Germany) with a length of 9 cm was fixed to the longitudinal ligament above the sacral promontory using two non-absorbable sutures (Ethibond 2-0, Johnson & Johnson) (Figure 3).

Thereafter a suture rectopexy fixed the perirectal peritoneal tissue of the rectum to the peritoneum on the left pelvic wall and the sacral vertebrae at the promontory with nonabsorbable sutures (Ethibond 2-0, Johnson & Johnson). At the end of the procedure the pelvic peritoneum was closed to obliterate the Douglas pouch and to cover the mesh structure on the right side with a suture non-absorbable suture (Ethibond 2-0, Johnson & Johnson) (Figure 3). According to our institutional standards, the patient received 1.5 g of cefazolin and 500 mg of metronidazole intraoperatively once.

## Results

The operation time was 223 minutes with no intraoperative complications. Blood loss was less than 100 ml. No postoperative complications occurred, and the patient was discharged 8 days after surgery.

Preoperative scores of the clinical bowel symptoms for ODS using the Altomare (7/30 vs. 0/30), bowel dysfunction using the rectal toxicity score (11/32 vs. 0/32), and faecal control using the Wexner incontinence score (14/20 vs. 2/20) improved after surgery. Anatomical outcome for POP improved from a POP-Q stage 2 prior to surgery to a POP-Q stage 0 after surgery (Table I). The stress urinary incontinence improved also.

## Discussion

The surgical treatment of ODS with POP aims at the anatomic reconstruction of the bowel and pelvic floor. So far, the treatment has been characterised by individual surgical approaches after the failure of conservative treatment options.

Surgical approaches for the medical condition of ODS accompanied by POP are characterised by a wide variety of individual approaches (Glazener et al., 2014; Grimes et al., 2016; Lee et al., 2020; Mattsson et al., 2020). An adequate treatment should address all aspects of the problem at once, however, a standardised option as in a combined interdisciplinary approach as a one-step approach for women with ODS and POP is missing (Wahed et al., 2012; Evans et al., 2015; McLean et al., 2018; Wei et al., 2019).

The presented surgical technique combines a laparoscopic approach between two disciplines, visceral surgery, and gynaecology, in an interdisciplinary surgical technique. The video demonstrates the combination of an RRP (with anastomosis and without a protective stoma) with a SCP (using a synthetic mesh) in one surgical procedure with no postoperative morbidity and good functional results.

Although the risk of anastomotic leakage might be seen as a risk factor for infectious mesh complication, the very low incidence in elective benign colorectal surgery below 5% and the advantage of a one- step approach with respect to overall morbidity is well balanced (Ambe et al., 2019; Maatouk et al., 2023). A rectopexy alone in cases of a relevant elongated left side colon may lead to a kinking of the colorectum with worsening of the defecation problems the approach intended to solve.

Furthermore, the SCP was performed with a minimum amount of synthetic material (mesh with a length of 9 cm and approximately 10 cm2). The mesh was attached to the vaginal vault and the longitudinal ligament at the promontory with three and two sutures each, respectively.

No postoperative morbidity occurred after surgery. Functional outcomes for bowel function and urinary incontinence were improved after surgery and an excellent anatomical outcome was achieved.

## Conclusions

The video study presents an interdisciplinary RRP with mesh- SCP in a combined surgical procedure as a one-stop treatment option for the complex medical condition of women suffering from ODS and POP. The procedure was feasible, safe, and effective in this case and should be considered as a treatment option for women with the underlying condition.

## Video scan (read QR)

https://vimeo.com/917027436/2c8497e170?share=copy

**Figure:**